# Mandibular reconstructions with free fibula flap using standardized partially adjustable cutting guides or CAD/CAM technique: a three- and two-dimensional comparison

**DOI:** 10.3389/fonc.2023.1167071

**Published:** 2023-05-09

**Authors:** Jochen Weitz, Alex Grabenhorst, Hannes Singer, Minli Niu, Florian D. Grill, Daniel Kamreh, Carolina A. S. Claßen, Klaus-Dietrich Wolff, Lucas M. Ritschl

**Affiliations:** ^1^ Department of Oral and Maxillofacial Surgery, Josefinum, Augsburg and Private Practice Oral and Maxillofacial Surgery im Pferseepark, Augsburg, Germany; ^2^ Department of Oral and Maxillofacial Surgery, School of Medicine, Technical University of Munich, Munich, Germany; ^3^ Department of Oral and Maxillofacial Surgery, School of Medicine, University of Saarland, Homburg, Saar, Germany

**Keywords:** mandibular reconstruction, three dimensional comparison, CAD/CAM planning, standardized partially adjustable cutting guides, free fibula flap

## Abstract

**Background:**

Mandibular reconstruction with the fibula free flap (FFF) is performed freehand, CAD/CAM-assisted, or by using partially adjustable resection/reconstruction aids. The two latter options represent the contemporary reconstructive solutions of the recent decade. The purpose of this study was to compare both auxiliary techniques with regard to feasibility, accuracy, and operative parameters.

**Methods and materials:**

The first twenty consecutively operated patients requiring a mandibular reconstruction (within angle-to-angle) with the FFF using the partially adjustable resection aids between January 2017 and December 2019 at our department were included. Additionally, matching CAD/CAM FFF cases were used as control group in this cross-sectional study. Medical records and general information (sex, age, indication for surgery, extent of resection, number of segments, duration of surgery, and ischemia time) were analyzed. In addition, the pre- and postoperative Digital Imaging and Communications in Medicine data of the mandibles were converted to standard tessellation language (.stl) files. Conventional measurements – six horizontal distances (A–F) and temporo-mandibular joint (TMJ) spaces – and the root mean square error (RMSE) for three-dimensional analysis were measured and calculated.

**Results:**

In total, 40 patients were enrolled (20:20). Overall operation time, ischemia time, and the interval between ischemia time start until end of operation showed no significant differences. No significant difference between the two groups were revealed in conventional measurements of distances (A–D) and TMJ spaces. The Δ differences for the distance F (between the mandibular foramina) and the right medial joint space were significantly lower in the ReconGuide group. The RMSE analysis of the two groups showed no significant difference (*p*=0.925), with an overall median RMSE of 3.1 mm (2.2–3.7) in the CAD/CAM and 2.9 mm (2.2–3.8) in the ReconGuide groups.

**Conclusions:**

The reconstructive surgeon can achieve comparable postoperative results regardless of technique, which may favor the ReconGuide use in mandibular angle-to-angle reconstruction over the CAD/CAM technique because of less preoperative planning time and lower costs per case.

## 1 Introduction

Mandibular primary or secondary reconstruction with the free fibula flap (FFF) has become a highly standardized procedure since its introduction. In the last decade, the integration and progress of virtual planning processes and three-dimensional (3D) printing have helped to increase intraoperative confidence. Consequently, the application of computer-aided design and computer-aided manufacturing (CAD/CAM) is considered to be state of the art nowadays as it leads to reduced operating and ischemic times and improves symmetry, bony consolidation, and function in microvascular mandibular reconstruction (1–3).

But usually the use of the CAD/CAM technique is associated with increased costs and a certain sort of dependency on a functioning infrastructure with nationwide coverage by various osteosynthesis manufacturers. This reduces planning flexibility and requires a lead time of seven to ten working days, during which one to three web meetings are held to discuss the virtual surgical plan and its implementation, except for centers that have their own software for this purpose.

Likewise, two different developments can be observed in the daily routine and more recent literature. First, the establishment of in-house virtual planning algorithms using open-source software and the production of cutting guides with in-house printers (4–8). Second, the development of partially adjustable resection/reconstruction aids such as the ReconGuide and the MUC-Jig (9, 10). ReconGuide is a special device that allows up to mandibular angle-to-angle reconstruction with up to three FFF segments by using a partially adjustable resection aid for the mandible and fibula to facilitate the fibula wedge osteotomies without the need of preoperative virtual planning. Nevertheless, the need for preoperative imaging of the arterial blood supply to the lower legs remains.

The purpose of this study was to evaluate and compare the results of the two mandibular reconstruction solutions with the FFF, namely the CAD/CAM technique or with the standardized partially adjustable resection/reconstruction aid system called ReconGuide, with regard to feasibility, accuracy, and operative parameters.

## 2 Material and methods

### 2.1 Ethical statement and patient recruitment

All clinical investigations and procedures were conducted according to the principles expressed in the Declaration of Helsinki. Written patient consent was obtained. This cross-sectional study was approved by the Ethical Committee of the Technische Universität München (Approval No. 459/18S-KK).

The first twenty consecutively operated patients requiring a mandibular reconstruction with the ReconGuide system between January 2017 and December 2019 at our department were included. Additionally, we screened our records to find the 20 closest-matching CAD/CAM FFF cases for this study.

### 2.2 Surgical procedure using the ReconGuide system

The application of the partially adjustable resection/reconstruction aid system ReconGuide (KLS Martin Group; Gebrüder Martin GmbH & Co. KG; Tuttlingen, Germany) is described elsewhere in detail (10). This device enables standardized resection between the mandibular angles, resulting in one-, two-, or three-segmented mandibular reconstructions of body, body-symphyseal, or body-symphyseal-body defects according to Urken et al. (11). Ramus, mandibular neck, or condylar head reconstructions have not been possible with this device yet.

**Figure 1:**
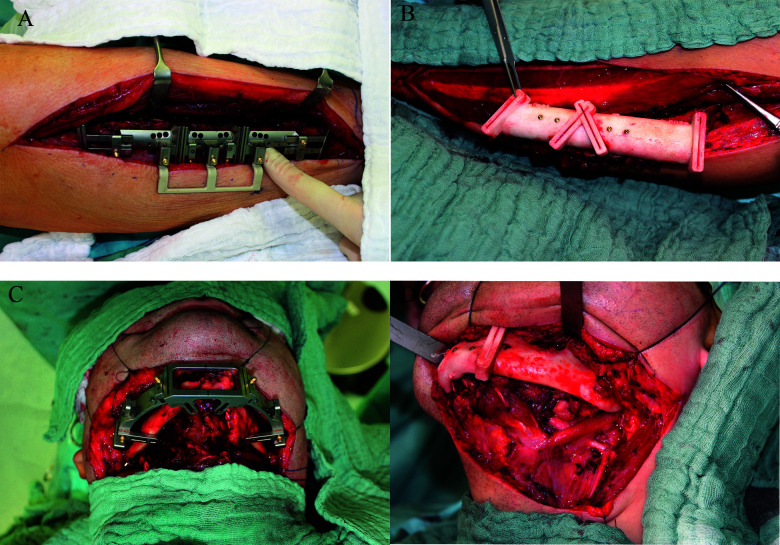
Intraoperative situation of both groups with fibular and mandibular resection aids *in situ* (**A**, **C**) ReconGuide system (KLS Martin Group; Gebrüder Martin GmbH & Co. KG; Tuttlingen, Germany) and (**B**, **D**) conventional CAD/CAM group.

The distance of resected corpus segments is adjustable within a length of 45–80 mm. The anterior symphyseal segment has a fixed and defined (outer = vestibular) length of 32 mm in order to guarantee the minimum required FFF bone length to ensure sufficient vascular supply (12). Corresponding lengths are transferred to the FFF partially adjustable resection aid (**Figure 1**). The osteotomy angles (parasymphyseal and angle region) are defined and not adjustable. The mandibular resection aid is placed *via* a conventional transcervical approach and the FFF aid is positioned on the lateral face of the fibula as known in conventional CAD/CAM solutions *via* a lateral approach.

### 2.3 CAD/CAM group

The CAD/CAM group consisted of defect-matched FFF cases that had been planned conventionally with osteosynthesis manufacturers (ex-house planning) at a time before our department acquired the ReconGuide system. In all these ex-house planned CAD/CAM cases 2.0 miniplates were used as osteosynthesis material. The manufacturer sent pre-bent and sterilized 2.0 miniplates, as well as mandibular and FFF CAD/CAM cutting aids.

### 2.4 Conventional measurements and three-dimensional analysis of postoperative results

**Figure 2:**
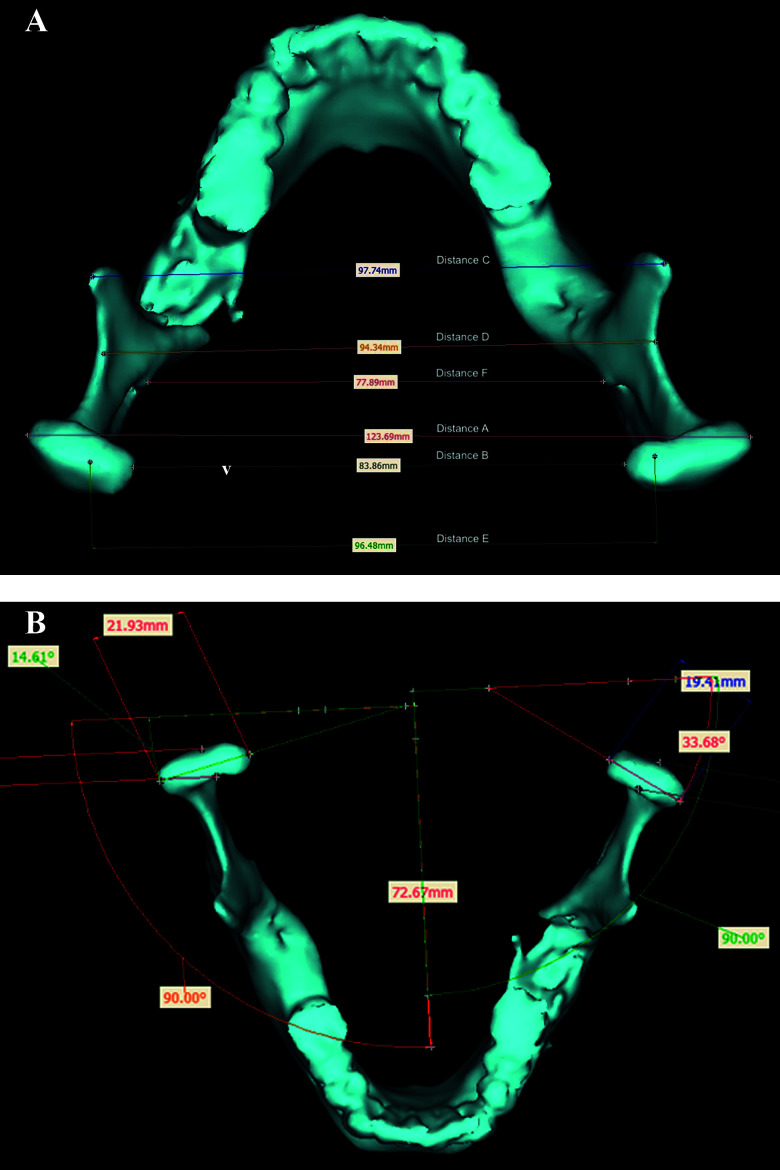
Overview of conventional measurements: **(A)** six horizontal distances between condylar head–condylar head (head–head) lateral (a) and medial (b), intercoronoidal (c), sigmoid notch (d), most cranial condylar head points (e), and mandibular foramina (f), and **(B)** calculation of the condylar head angle left and right.

**Figure 3:**
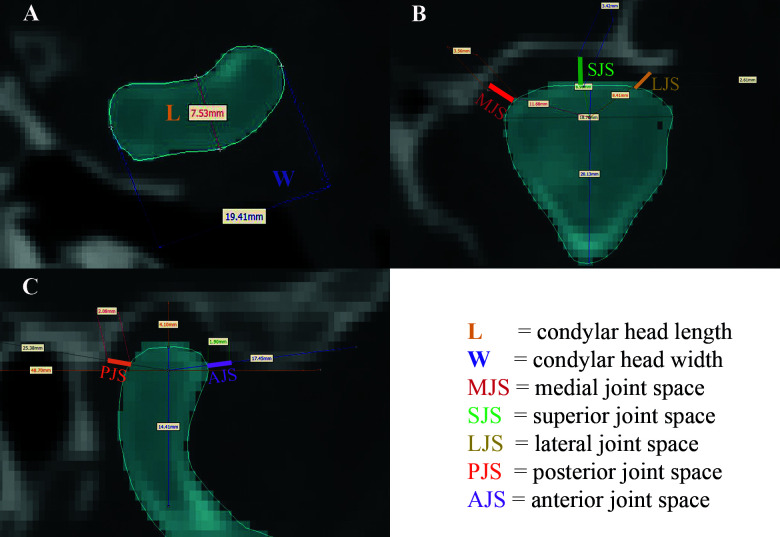
**(A)** Measurement of condylar width (W) and length (L); **(B)** measurement of medial, superior, and lateral joint spaces (MJS, SJS, and LJS); **(C)** measurement of anterior and posterior joint spaces (AJS and PJS).

Postoperative metric analysis of the reconstructed mandibles included conventional measurements of distances and angles, and 3D surface matching methods. In a first step the corresponding pre- and postoperative CT-based Digital Imaging and Communications in Medicine (DICOM) data sets were converted into standard tessellation language (.stl) mandible models using Mimics^®^ software (Mimics^®^ 17.0, Materialise; Leuven, Belgium). The conventional parameters were then measured at the segmented mandibles, including the horizontal distances between condylar head–condylar head (head–head) lateral (A) and medial (B) border, intercoronoidal distance (C), between the sigmoid notches (D), between the most cranial condylar head points (E), and between both mandibular foramina (F) (**Figure 2A**). Additionally, the condylar head width, length, and angles left and right as well as five temporo-mandibular joint (TMJ) spaces (anterior, lateral, medial, posterior, and superior) according to Ueki et al. (13) were measured (**Figures 2B**, **3A–C**).

**Figure 4:**
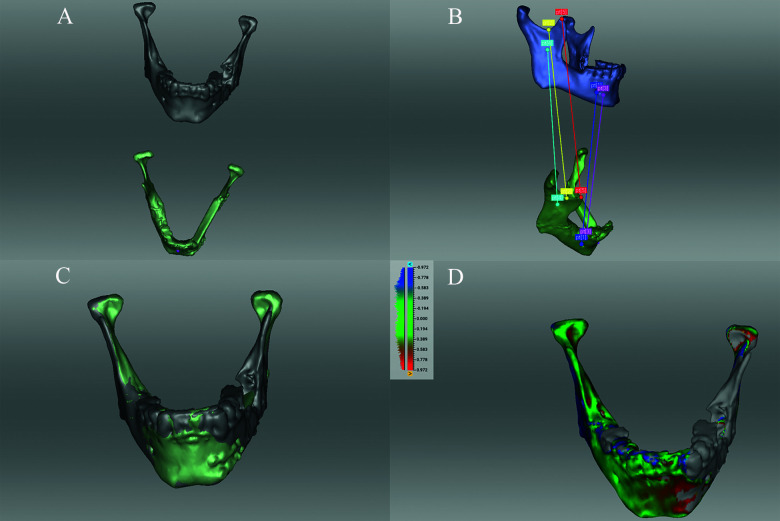
Three-dimensional surface matching procedure of the corresponding pre- and postoperative.stl models after import to Artec software (Artec Studio 13 Professional x64; Artec^®^ Group; Luxembourg): **(A)** imported corresponding pre- and postoperative.stl models; **(B)** six-point-aligned; **(C)** matching control, and **(D)** calculation of root mean square error (RMSE in mm).

Lastly, we performed a 3D surface matching procedure of the corresponding pre- and postoperative.stl models in order to objectively quantify the difficult parameter *accuracy* (3, 8). For this purpose the files were exported and six-point-aligned using Artec software (Artec Studio 13 Professional x64; Artec^®^ Group; Luxembourg) to calculate the root mean square error (RMSE, [mm]) (**Figures 4A–D**) (14, 15).

### 2.5 Statistical analysis

For the analysis of pre- and postoperative differences of the conventional parameters the Wilcoxon signed-rank test was used. The Mann–Whitney U test was used to compare operative and postoperative parameters between the two groups. For the paired testing between pre- and postoperative comparison of the conventional parameters, the Wilcoxon test was used. Uni- and multivariate regression analyses were performed for the differences of RMSE between pre- and postoperative models.

All statistical tests were performed on an exploratory two-sided 5% significance level. No adjustments were made for multiple testing. Analysis was done with IBM SPSS 24 for Mac software (IBM Corp, Armonk; New York, United States).

## 3 Results

### 3.1 General parameters

General information of the enrolled patients is shown in **Table 1**. Regarding defect size according to Brown et al., the number of segments (*p*=0.774) as well as the way of raising free fibula as an osteomyocutaneous or osseous flap showed no significant difference (*p*=0.232 and *p*=0.637, respectively). No secondary reconstructions or osteomyelitis cases were operated in the ReconGuide group.

**Table 1:** Overview of enrolled patients with regard to registered parameters: Gender, age, indication for surgery, free fibula flap architecture, and mandibular defect class according to Brown et al. (16).

Parameters	CAD/CAM (n=20)		ReconGuide (n=20)	
**Gender f/m**	7/13		9/11	
**Age median (range)**	60 (30–82)		64 (34–85)	
**Indication**	OSCC ORN MRONJ Other malignancy 2° Reconstruction Osteomyelitis	10 2 1 5 1 1	OSCC ORN MRONJ Other malignancy 2° Reconstruction Osteomyelitis	10 5 5 0 0 0
**Free fibula flaps**	Osteomyocutaneous Osseous	17 3	Osteomyocutaneous Osseous	18 2
**Mandibular defect**	I II IV Ic IIc	4 9 4 1 2	I II IV	4 9 7

OSCC, oral squamous cell carcinoma; ORN, osteoradionecrosis; MRONJ, medication-related osteonecrosis of the jaw; 2° secondary.

Overall operation time was faster in the ReconGuide group (599 minutes (328–907); *p*=0.213), whereas both ischemia time and the interval beginning of ischemia time until end of operation were shorter in CAD/CAM group (115 minutes (60–220) (*p*=0.491) and 280 minutes (160–472) (*p*=0.580) respectively (**Table 2**)). Median duration of hospital stay was 13 days (10–45) for the CAD/CAM group and 21 days (10–44) for the ReconGuide group but showed no significant difference (*p*=0.051).

**Table 2:** Comparison of operative and postoperative parameters between the two groups: CAD/CAM (n=20) versus ReconGuide (n=20).

Parameters	CAD/CAM	ReconGuide	*p*-value ^#^
**Operation time [min]**	650 (438–901)	599 (328–907)	0.213
**Ischemia time [min]**	115 (60–220)	139 (56–224)	0.491
**Ischemia time – end of operation [min]**	280 (160–472)	307 (190–513)	0.580
**Incidence of revision**	0	0	1.000
**Free flap loss**	0	0	1.000

Min, minutes; CAD/CAM, computer-aided design and computer-aided manufacturing.

Median (range).

^#^Mann–Whitney U test.

### 3.2 Measurements – conventional and root mean square error analysis

The results of conventional measurements of distances (A–F) and TMJ spaces right/left as well as condylar head length, width, and angles right/left, including the preoperative situation and the differences between the pre- and postoperative result (Δ difference), are displayed in **Table 3**. No significant differences between the two groups were revealed, except for the distance F (distance between the mandibular foramina) and the right medial joint space, where the ReconGuide group showed significantly lower Δ differences than the CAD/CAM group (*p*=0.023 and *p*=0.016, respectively, **Table 3**).

**Table 3:** Comparison of conventional measurements of the differences (Δ = preoperative vs. postoperative) between the two groups: CAD/CAM (n=20) vs. ReconGuide (n=20).

Parameters	CAD/CAM		ReconGuide		*p*-value ^#^
	*Preoperative result*	*Δ Difference*	*Preoperative result*	*Δ Difference*	
**Distance A**	121.9 (111.4–128.4)	1.5 (-1.8–7.7)	123.1 (106.0–132.8)	1.1 (-3.9–20.9)	0.229
**Distance B**	84.5 (74.7–92.9)	1.0 (-4.8–7.8)	83.9 (71.7–89.9)	0.2 (-4.8–2.9)	0.144
**Distance C**	99.4 (88.9–105.2)	1.6 (-9.6–3.7)	96.8 (90.8–109.7)	2.3 (-9.6–11.1)	0.160
**Distance D**	97.7 (90.1–104.7)	1.3 (-2.7–4.3)	97.6 (87.6–105.6)	0.1 (-5.6–5.0)	0.540
**Distance E**	98.2 (80.7–112.4)	-0.2 (-4.0–12.2)	96.2 (86.7–106.7)	0.9 (-11.0–17.3)	0.762
**Distance F**	82.5 (73.0–93.1)	5.0 (-2.2–7.6)	82.3 (75.3–97.1)	-0.2 (-4.7–13.3)	0.023
**Right ant. joint space**	2.5 (1.1–3.8)	-0.2 (-5.8 –0.8)	1.9 (0.7–2.9)	-0.4 (-2.4–0.8)	0.433
**Right lat. joint space**	3.1 (1.5–5.3)	-0.1 (-2.0–1.2)	2.4 (0.9–5.4)	-0.1 (-3.7–2.1)	0.804
**Right med. joint space**	2.9 (1.9–5.6)	0.8 (-0.5–2.4)	2.5 (1.0–5.3)	0.1 (-2.1–1.0)	0.016
**Right post. joint space**	2.8 (1.7–4.5)	-0.1 (-9.0–1.6)	2.3 (1.5–4.3)	0.2 (-6.7–1.7)	0.932
**Right sup. joint space**	2.9 (1.9–5.2)	-0.8 (-9.7–1.0)	2.6 (0.7–4.0)	-0.3 (-3.8–1.7)	0.736
**Right condylar head length**	7.7 (5.8–10.6)	0.0 (-0.6–0.7)	8.4 (1.0–11.9)	0.0 (-0.5–0.7)	0.791
**Right condylar head width**	19.9 (9.6–22.2)	0.0 (-1.4–0.5)	21.2 (16.4–23.5)	0.1 (-1.2–1.1)	0.560
**Left ant. joint space**	2.2 (1.4–3.9)	0.2 (-2.0–1.5)	2.0 (1.2–3.4)	-0.5 (-2.2–0.6)	0.171
**Left lat. joint space**	2.8 (1.5–4.3)	-0.3 (-4.5–2.6)	2.2 (0.8–4.1)	-0.1 (-2.6–0.4)	0.281
**Left med. joint space**	3.3 (2.2–5.3)	0.0 (-1.3–1.8)	2.7 (1.6–5.2)	-0.2 (-3.9–3.0)	0.098
**Left post. joint space**	2.7 (1.7–5.7)	-0.3 (-6.8–3.1)	2.3 (0.8–3.5)	-0.2 (-6.6–1.6)	0.702
**Left sup. joint space**	2.9 (1.4–4.2)	-0.3 (-5.5–1.3)	2.4 (0.9–4.1)	-0.3 (-3.2–0.5)	0.757
**Left condylar head length**	8.0 (6.5–11.1)	0.0 (-0.1–0.5)	8.7 (6.7–11.3)	0.0 (-0.3–1.0)	0.864
**Left condylar head width**	20.3 (16.9–23.4)	0.1 (-0.1–2.3)	21.1 (15.3–24.5)	0.1 (-0.7–1.7)	0.907
**Condylar head angle right [°]**	23.6 (10.5–39.5)	-1.8 (-16.5–9.9)	24.6 (5.5–31.5)	0.8 (-13.1–10.7)	0.632
**Condylar head angle left [°]**	26.0 (14.4–35.0)	0.7 (-26.5–14.7)	24.1 (15.4–36.2)	-1.5 (-11.6–11.4)	0.350

CAD/CAM, computer-aided design and computer-aided manufacturing; ant., anterior; lat, lateral; med., medial; post., posterior; sup., superior.

Median (range).

^#^Mann–Whitney U test between Δ difference between the two groups.

Comparing pre- and postoperative conventional parameters in the CAD/CAM group, the left lateral joint space (*p*=0.049) and the right medial joint space (*p*=0.025) changed significantly. Comparing pre- and postoperative conventional parameters in the ReconGuide group, the left and right anterior joint spaces (*p*=0.016 and *p*=0.013, respectively), left superior joint space (*p*=0.010), and left condylar head angle (*p*=0.030) changed significantly. None of the analyzed distances A–F showed any significant pre- to postoperative change within one group.

Three-dimensional surface matching applying the RMSE analysis to both groups showed no significant difference (*p*=0.925). Overall median RMSE was 3.1 mm (2.2–3.7) for the CAD/CAM group and 2.9 mm (2.2–3.8) in the ReconGuide group. The results for segment-vice comparison of the two groups are displayed in **Table 4** and also remained without a significant difference between the two groups.

**Table 4:** Three-dimensional surface matching applying the root mean square error analysis (RMSE in mm) between the two groups: CAD/CAM (n=20) vs. ReconGuide (n=20).

Parameters	CAD/CAM	ReconGuide	*p*-value ^#^
**Overall**	3.1 (2.2–3.7)	2.9 (2.2–3.8)	0.925
**One-segmented FFF**	2.9 (2.6–3.3)	2.5 (2.2–3.4)	0.564
**Two-segmented FFF**	2.6 (2.2–3.4)	2.9 (2.3–3.6)	0.556
**Three-segmented FFF**	3.1 (2.2–3.7)	3.0 (2.3–3.8)	0.758

CAD/CAM, computer-aided design and computer-aided manufacturing.

Median (range).

^#^Mann–Whitney U test.

## 4 Discussion

With this study we could not demonstrate many significant differences in the way the mandibular reconstruction was performed with regard to the different operation times and metric parameters investigated. This is currently the only study that compares ex-house CAD/CAM-assisted mandibular reconstruction with the partially adjustable ReconGuide system in a comparative pre- and postoperative fashion. Nobis et al. recently demonstrated no superiority of any osteosynthesis material in mandibular reconstructive techniques (CAD/CAM vs. freehand vs. ReconGuide) regarding complications and bony consolidation rates (17). In our study, all plates were pre-bent 2.0 miniplates in both groups, as the ReconGuide method offers osteotomy-specific pre-bent 2.0 miniplates. Based on our experience and to reduce a selection bias due to osteosynthesis (18), we only included CAD/CAM cases with pre-bent 2.0 miniplates. Both, (pre-bent) miniplates and (patient specific) reconstruction plates, have pros and contras, which are ongoing discussed. Several points that have to be addressed, when you discuss this topic: biomechanics, stiffness of fixation, bone healing and bony junction, impairment of blood supply due to fixation system, implant insertion for oral rehabilitation. Osseous fixation with pre-bent miniplates offers many advantages, even though it needs nearly perfect alignment of the bony interface, resulting in a very low (critical) osteotomy gap distance (19). Using a reconstruction plate, the role and influence of the bony gap decreases because of the biomechanical stiffness of the plate. A recent retrospective analysis has shown more failed bone junction in the mor rigid PSI group (20) and the physiological chewing forces are withdrawn and inhibit physiological bone remodeling. Using miniplates, the forces seem to stimulate bone healing which can be seen in additional bone volume at the bone junction sides of the FFF (21) and are therefore from a mechanobiological point of view beneficial (22). The latest development in this field are patient specific miniplates and the development of this modifications will be interesting to follow (22). When it comes to dental implant insertion, miniplate removal prior to implantation is possible in local anesthesia within the implantation. If a (patient specific) reconstruction plate has been used for osteosynthesis, it has to be removed only partially *via* an intraoral approach or *via* reopening the neck under general anesthesia (resulting in anesthesiologic and surgical risks and higher costs for the health system) (23).

Essentially, this study was about time (overall surgical, ischemia, and reconstruction time), as well as metric analyses of defined and established distances (13). In addition, we performed a 3D matching of the segmented corresponding pre- and postoperative mandibles and calculated the RMSE. This method of comparison and analysis is in our eyes an appropriate way to evaluate the real three-dimensional anatomical success of the surgery, as we described before (8). Furthermore, it is still not defined in the literature what exactly is meant by “accuracy” in the context of mandibular reconstruction. One critical parameter would be a stable occlusion, which however has not been evaluated in this study, is rarely analyzed in other studies, and is impossible to use as a key reference in complete angle-to-angle reconstructions. Essentially, in our view, anatomical accuracy is reflected by the fact that there is no significant difference postoperatively from the preoperative baseline situation, resulting in a low RMSE. This is present here in both groups, representing a good surgical quality and result. Incorrect virtual planning or inappropriate use of the ReconGuide system would have led to a derotation of the mandibular ramus and condylar head, and consequently change the condyle angle and position. Additionally, the RMSE result would have been higher. But our RMSE results were comparable to other studies that have used this parameter (5, 8, 24). Hence, in a metaphorical sense, the off-the-peg suit achieved an equivalent anatomical accuracy result to the made-to-measure suit in this study.

But one of the main conclusions of this comparative study is that the less time-consuming and less expensive method can compete very well in the quality of the reconstruction and is by no means inferior. The prefabricated 2.0 miniplates fit very well, especially paramedian and usually also at the mandibular angle region. The acquisition cost of the ReconGuide system is approximately EUR 10,000 (instrument set and resection aids) or EUR 18,000 (instrument set, resection aids, and set of prefabricated miniplates). The cost of CAD/CAM planning in the way we did it was about EUR 3,000 per case. Thus, the purchase of the ReconGuide system paid for itself after only six cases. Only the in-house planning and printing of resection aids as described above would be cheaper (5, 8, 24). But here again, the time required for in-house planning has a negative effect on this technique as it requires more than just commitment and there are no standardized or applicable compensation models yet.

A negative aspect of the ReconGuide system is that the preoperative active teaching of younger surgeons is eliminated by replacing in-house or ex-house planning with the necessary virtual planning meetings and discussions. How this in turn will affect training is currently unclear and unpredictable.

### 4.1 Limitations

One limitation of this study was the use of license-based segmentation software, which is associated with an additional acquisition. More recently, open-source segmentation solutions like Slicer have been shown to be reliable and reproducible (25–27). But we used license-based segmentation software in order to reduce potential software-based errors. The postoperative interval of imaging was short. This does not allow any conclusions about the long-term stability of mandibular ramus and condylar head position as changes may also occur later in a longer observational interval than was the case in our study (28). But nevertheless, a derotation of the condylar head because of cranial ramus rotation will be visible immediately. The functional sequalae is uncertain and needs further evaluation (29).

Last, we did not use oral rehabilitation or occlusion as the main parameter in this study for several reasons, even though it would represent the final goal of microvascular mandibular reconstruction. One reason is the focus of the ReconGuide system on anatomical restoration of the mandible, which does not allow any kind of backward planning for optimized oral dental implant placement for the final full or partial arch prosthesis. This would have resulted in a selection bias. A second reason is that by initiating this study no objective analysis and capture of the preoperative occlusion has been recorded for scientific purposes and would have consequently reduced the study cohort. So as a first critical step we have focused in this study on the purely anatomical restoration of mandibular continuity.

## 5 Conclusions

Nowadays mandibular reconstruction is a very standardized procedure with good postoperative results regarding symmetry and accuracy. Based on the interesting question of how much help and planning is necessary, this study compares two methods, namely the CAD/CAM technique and the partially adjustable resection/reconstruction aid system called ReconGuide. Within the limits of this study, the reconstructive surgeon can achieve comparable postoperative results regardless of technique, which may favor the use of ReconGuide in angle-to-angle reconstruction over the CAD/CAM technique because of less preoperative planning time and lower costs per case, especially for regions with fewer financial opportunities and cases for which no backward planning is provided.

## Data availability statement

The original contributions presented in the study are included in the article. Further inquiries can be directed to the corresponding author.

## Ethics statement

The studies involving human participants were reviewed and approved by Ethical Committee of the Technische Universität München (Approval No. 459/18S-KK). The ethics committee waived the requirement of written informed consent for participation.

## Author contributions

JW: Study design/conduction, operations, data interpretation, and major contribution to manuscript writing and revision AG: Data acquisition and interpretation, contribution to revision. HS: Creation of figures, statistical analysis, contribution to revision. MN: Statistical analysis, data interpretation, contribution to revision. FG: Study design/conduction, operations and contribution to revision DK: Surface matching and analysis, data acquisition and interpretation, statistical analysis. CC: Data interpretation, creation of tables, contribution to revision K-DW: Study design/conduction, operations, data interpretation, contribution to revision. LR: Study design/conduction, operations, data interpretation, and major contribution to manuscript writing. All authors contributed to the article and approved the submitted version.
